# H-Type Hypertension and C Reactive Protein in Recurrence of Ischemic Stroke

**DOI:** 10.3390/ijerph13050477

**Published:** 2016-05-07

**Authors:** Qing Zhang, De-Xing Qiu, Rong-Li Fu, Tian-Fen Xu, Meng-Juan Jing, Hui-Shan Zhang, He-Hong Geng, Long-Chao Zheng, Pei-Xi Wang

**Affiliations:** 1 Institute of Public Health, School of Nursing, Henan University, Kaifeng 475004, China; zq71200@163.com (Q.Z.); jing53905@163.com (M.-J.J.); 15226003150@163.com (H.-H.G.); 2 Guangming New District People’s Hospital & Community Health Service Management Center of Guangming Area, Shenzhen 518000, China; qiudexing66@139.com; 3 Internal Medicine-Neurology, Huaihe Hospital, Henan University, Kaifeng 475000, China; 15225475536@163.com; 4 Basical School, Guangzhou Medical University, Guangzhou 510180, China; xutianfen01@sina.com; 5 Department of Preventive Medicine, School of Public Health, Guangzhou Medical University, Guangzhou 510180, China; zhanghuishan25@163.com; 6 Department of Public Health, School of Public Health, Graduate School of Guizhou Medical University, Guiyang 550025, China; zlc3050@163.com

**Keywords:** C reactive protein, H-type hypertension, recurrence ischemic stroke

## Abstract

Hypertension with high homocysteine (HHcy) (H-type hypertension) and C reactive protein (CRP) can increase the incidence of ischemic stroke. However, it is not clear whether recurrent ischemic stroke (RIS) is related to H-type hypertension and CRP. The present study investigated the correlation of H-type hypertension and CRP level with RIS. Totally, 987 consecutive patients with acute ischemic stroke were recruited in a teaching hospital in Henan province, China during March 2014 to March 2015. The demographic and clinical characteristics and blood biochemical parameters of patients were analyzed. Elevated levels of CRP and homocysteine (Hcy) were defined as >8.2 mg/L and 10 μmol/L, respectively. Among the 987 patients, 234 were RIS. Thirty-eight percent of RIS patients had elevated CRP level and 91.5% of RIS patients had HHcy. In multivariate analysis, adjusted odds ratio (OR) of RIS in patients aged ≥60 years was 1.576 (95% CI: 1.125–2.207), in male patients 1.935 (95% CI: 1.385–2.704), in patients with diabetes 1.463 (95% CI: 1.037–2.064), CRP levels 1.013 (95% CI: 1.006–1.019), simple hypertension 3.370 (95% CI: 1.15–10.183), and H-type hypertension 2.990 (95% CI: 1.176–7.600). RIS was associated with older age, male, diabetes, H-type hypertension and CRP. Controlling H-type hypertension and CRP level may reduce the risk of RIS.

## 1. Introduction

Cerebrovascular disease has become the leading cause of death in China, having about 60%–80% attribution by ischemic stroke [1], which is also one of the main causes of death in many countries. With the improvement of medicine, the mortality rate of stroke has declined, but the recurrence rate is increasing in recent years. Previous research showed that the risk of recurrent ischemic stroke (RIS) reaches up to 9% after six months, with cumulative rate about 4% after one year and 10-year recurrence rate of 43% [2]. RIS demonstrates higher mortality and disability rate than the first-ever ischemic stroke (FIS), and it also causes more economic pressure and manpower burden to families and society.

Previous studies showed that many risk factors are related to the RIS, including atherosclerosis, hypertension, diabetes, hyperlipidemia, smoking, drinking and so on [3,4,5,6]. Recently, the association between serum biochemical parameters and stroke has received more attention. There is a noticed characteristic in Chinese that about 75% of Chinese hypertensive patients are accompanied by high homocysteine (HHcy) [7], so the concept of H-type hypertension (hypertension associated with HHcy) was proposed [8]. For stroke risk, hypertension and HHcy have shown a multiplicative effect. Previous studies found that H-type hypertension increases the risk of FIS [9]. C-reactive protein (CRP) is produced mainly by hepatocytes and is regulated by inflammatory cytokines including interleukin-6 (IL-6) and tumor necrosis factor-α (TNF-α) [10]. Previous studies indicated that increased level of CRP can confirm the presence of active atherosclerotic lesions [11] and is also closely related to FIS [12]. However, the relationship between CRP and RIS is not clear.

The purpose of this study is to compare the differences of Hcy and CRP levels in patients with FIS and RIS, and to explore the relationship between H-type hypertension and CRP levels and RIS.

## 2. Materials and Methods

### 2.1. Study Subjects

**Figure 1:**
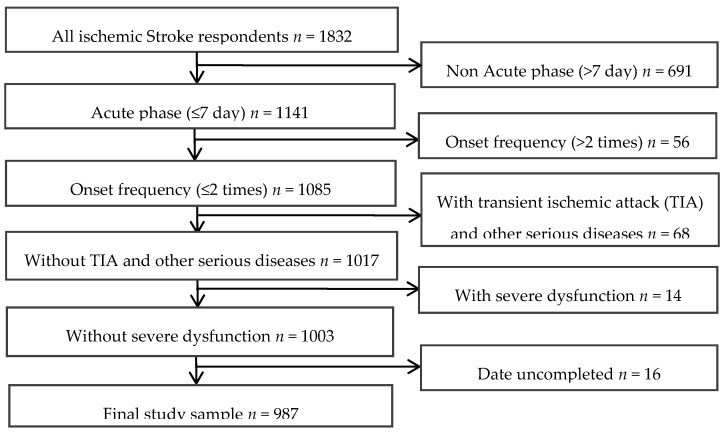
Flow chart in the selection of study patients.

The data of 987 consecutive patients who were hospitalized during acute phase (the first week following a stroke) in Huai-He Hospital, Kaifeng, China from March 2014 to March 2015 were analyzed with cross-sectional observational study. All patients signed the informed consent form. All patients were diagnosed with ischemic stroke and confirmed by MRI and/or CT according to the fourth Chinese National Conference’s recommendations on the diagnosis of cerebrovascular diseases [13]. RIS patients included 234 cases (163 male and 71 female), FIS patients included 753 cases as the control group (432 male and 321 female). Inclusion criteria for the study were >18 years of age, and no serious consciousness and cognitive impairment. The serum CRP and Hcy levels were measured within 24 h of hospitalization. The exclusion criteria were hemorrhagic stroke, transient ischemic attack (TIA), Parkinson’s disease, posterior circulation ischemia, epilepsy, subarachnoid hemorrhage, recent surgery, trauma or acute infection and other significant acute medical illness (e.g., serious heart, lung, liver, kidney disease or malignant tumor) and onset frequency >2 times. A flowchart illustrating the selection of study patients was presented in Figure 1.

#### 2.1.1. Socio-Demographic and Lifestyle Variables

Socio-demographic variables including age, gender, marital status and place of residence (urban or rural) and lifestyle factors including smoking status, alcohol drinking status, family history, hypertension history, diabetes history, coronary heart disease history and hyperlipidemia were analyzed. Smoking was defined as having smoking one or more cigarettes per day for one year, or smoking cessation <5 years [14]. Alcohol drinking was defined as drinking ≥1 time/week for one or more years. Family history of ischemic stroke was defined as having a history of ischemic stroke in first to third degree relatives. Hyperlipidemia was defined as having elevated plasma level in at least one of total cholesterol, triglyceride and low density lipoprotein. Hypertension was defined as having previous history of hypertension, systolic blood pressure > 140 mmHg and/or diastolic blood pressure > 90 mmHg for two times in the quiet condition in hospital for patients with hypertension history, in addition to, even if the blood pressure is not higher than 140/90 mmHg for patients taking antihypertensive drugs, also known as hypertension patients. Diabetes was defined as having previous history of diabetes, at the same times, fasting blood glucose > 7.0 mmol/L after admission or postprandial 2 h plasma glucose > 11.1 mmol/L or be clearly diagnosed as diabetes by glucose tolerance test or patients without diabetes history, also known as diabetes patients.

#### 2.1.2. Blood Collection and Laboratory Test

The fasting venous samples were collected within 24 h of hospitalization. Venous blood samples from patients after an 8 ± 10 h fast were collected in tubes containing EDTA and centrifuged immediately. The obtained plasma was kept at −20 °C. The level of serum Hcy was detected using high-performance liquid chromatography (HPLC) according to the manual. H-type hypertension was defined as Hcy level ≥ 10 μmol/L. This cutoff was selected at a level ≥ 10 μmol/L in accordance with previous studies [15,16].

The patients with the increase of Hcy only were defined the simple HHcy group, and the patients with elevated blood pressure only were defined the simple hypertension group. The individuals with neither high blood pressure nor HHcy were defined normal group. Serum CRP level was detected using immune assay according to the manual. The level of CRP > 8.2 mg/L was defined as high CRP.

### 2.2. Statistical Analysis

The statistical analysis was conducted with SPSS13.0 (SPSS, Inc., Chicago, IL, USA). Continuous variables were expressed as mean ± SD (Standard Deviation) while categorical variables were expressed as frequency and percentage. The data were proved for normal distribution by the Kolmogorov–Smirnov test. Homogeneity of variance was tested using Levene’s test. Two-tailed analyses were performed with the level of significance set at *p* < 0.05. Odds ratios (OR) with 95% confidence intervals are presented. The differences in characteristics of FIS and RIS were analyzed using *t*-test or χ^2^-test. Multivariate logistic regression was applied to investigate the risk factors of RIS. There was no significant colinearity between predictor variables affecting the stability of the regression models. *p* < 0.05 was set as significant level.

## 3. Results

### 3.1. General Characteristics of Subjects

In this study, a total of 987 patients with ischemic stroke in acute phase (onset frequency ≤ 2 times) were included and the general characteristics were listed in Table 1. Among these factors, elder age, male, living in urban, smoking, diabetes history and hyperlipidemia history were significant different between RIS and FIS (*p* < 0.05). There was no significant difference between RIS and FIS in marital status, smoking, alcohol drinking, family history, coronary heart disease, glucose (GLU), uric acid (UA), fibrinogen (FIB), white blood cells (WBC), red blood cells (RBC), platelets (PLT) and Hcy level (*p* > 0.05).

### 3.2. The Effect of CRP, Hypertension and HHcy on the Incidence of RIS

As shown in Table 1, the level of CRP in RIS group (16.94 ± 5.08 mg/L) was significantly higher than that in FIS group (8.11 ± 1.18 mg/L) (*t* = 3.334, *p* = 0.001). In addition, 321 patients had high level of CRP, and 89 of them were in RIS group (27.7%); 666 patients had normal CRP levels, 145 of them were in RIS group (21.8%). The prevalence of RIS in patients with high CRP level was a risk factor for RIS and there was significant difference in the incident of RIS between high CRP group and normal CRP group (χ^2^ = 4.245, *p* = 0.046).

Of all patients, 881 patients had HHcy, including 214 patients in RIS group and 667 patients in FIS group. The incidence of RIS and FIS in patients with HHcy levels was significantly higher than individuals with normal Hcy levels, but there was no significant difference in HHcy level between FIS and RIS groups (χ^2^ = 1.538, *p* = 0.229).

In all 987 patients, 158 patients had RIS in 564 patients with hypertension, while 76 patients had RIS in 423 patients without hypertension. The incidence of RIS in patients with hypertension (28%) was significantly higher than patients without hypertension (18%) (χ^2^ = 13.490, *p* < 0.001). The incidence of FIS was not significant between hypertension and non-hypertension groups (72% *vs.* 82%, *p* > 0.05).

Furthermore, the analysis of the relationship of H-type hypertension with RIS indicated that H-type hypertension was closely correlated with RIS (χ^2^ = 14.989, *p* = 0.002; Table 2).

### 3.3. Multivariate Logistic Regression Analysis

A number of independent predictors of RIS were shown in multivariate logistic regression analysis (Table 3). After the multivariate adjustment, RIS in acute phase was significantly related to male (OR = 1.935, 95% CI: 1.385–2.704), the elderly (OR = 1.576, 95% CI: 1.125–2.207), diabetes (OR = 1.463, 95% CI: 1.037–2.064), high CRP levels (OR = 1.013, 95% CI: 1.006–1.019), simple hypertension (OR = 3.370, 95% CI: 1.115–10.183), and H-type hypertension (OR = 2.990, 95% CI: 1.176–7.600). The associations with urban living and having hyperlipidemia were not statistically significant and therefore not included in the final parsimonious regression model.

## 4. Discussion

In the present study, more than one-third of patients with RIS were diagnosed with elevated CRP, and almost all patients with RIS were diagnosed with HHcy. The level of CRP and incidence of H-type hypertension in RIS was significantly higher than that in FIS. These results suggested that CRP and H-type hypertension were independent risk factors for RIS. Unexpectedly, the level of Hcy had no significant effect on the incidence of RIS, which was mainly caused by the significant impact of hypertension.

Previous research observed that older age [17,18], gender [19,20,21] and diabetes [22,23,24,25] were closely related to RIS. Consistently, the present study found that older age, male and having diabetes were independent risk factors for RIS after adjusting for covariates. The living habits such as effect of smoking and drinking, and the more physical work and life pressures in men may be partial explanation of the effect of gender on higher RIS, which remains to be further studied. Diabetes might increase the risk of stroke recurrence through endogenous cardiovascular NO system [26,27] while hyperglycemia may disrupt the blood–brain barrier and promote hemorrhagic infarct conversion [28]. However, the biological mechanism of effects of diabetes on RIS is not clear.

Inflammation is the nature in atherosclerosis, and CRP is an inflammation marker in the prediction of cardiovascular events [29]. Relatively few studies have corroborated CRP as a risk factor of ischemic stroke, mainly focused on FIS [30,31,32]. Previous studies indicated that CRP is a strong but nonspecific risk factor of fatal stroke in elder Europeans [33] and that the increased level of CRP is associated with a worse outcome in patients with ischemic stroke [34]. The present results indicated that the level of CRP in RIS was significantly higher than that in FIS and that the CRP level was an independent risk factor for RIS after adjusting for covariates (OR = 1.012, 95% CI: 1.006–1.018), which is similar with previous study suggesting that hsCRP predicts the risk of RIS among patients with recent lacunar stroke [35]. The mechanism of correlation of high CRP with RIS is currently unclear. Some studies indicated that there was a significant relationship between elevated CRP and atherosclerosis [36,37] while other studies showed that CRP was not associated with large atherosclerosis [38,39]. Aspirin can prevent the development of vascular endothelial dysfunction in experimental inflammation [40] and reduce the incidence of cardiovascular events in patients with an elevated CRP [41], suggesting that CRP may mediate the inflammation during RIS. More studies will be needed to clarify the relationship between CRP and RIS in future.

Previous studies indicated that hypertension and HHcy are the two most important risk factors for stroke [42,43] and associated with RIS [44,45,46]. In the present study, we found that hypertension was remarkably correlated with RIS but, surprisingly, no association between HHcy and RIS was verified although the average level of Hcy in FIS group and RIS group were higher than normal (10 μmol/L). In recent years, H-type hypertension has become a research hot-topic. Graham *et al.* found that HHcy and hypertension had a certain synergy in the occurrence of cardiovascular and cerebrovascular diseases, and the incidence of cardiovascular events in patients with H-type hypertension was about five times higher than that of patients with simple high blood pressure [47]. Mi *et al.* found synergistic effect of hypertension and Hcy in senile recurrence stroke [48]. The present study found that H-type hypertension (OR = 2.988, 95% CI: 1.162–7.686) was an independent risk factor for RIS, which was mainly because of the significant impact of hypertension (OR = 3.263, 95% CI: 1.067–9.980) on RIS. The difference in the findings might be due to the sample size and the selection of the control group. In addition, patients with HHcy in the hospital were told to take folic acid and other drugs after discharge, which may affect the results and remains to be further studied because it was found that supplement of folate reduced serum Hcy levels [49] and showed protective effect on stroke [50].

## 5. Limitations of the Study

Our study is to investigate the correlation between H-type Hypertension and CRP and recurrence of ischemic stroke. However, some limitations exist in our study. This study did not include other information (severity of ischemic stroke, recovery rate, and MRI/CT data) for analysis. Furthermore, the study is a cross-sectional design, and such causality could not be accurately deduced, and a larger and more prospective cohort study is needed to confirm the study results. Subsequently, some of FIS in our study could become RIS in future, and we will also conduct a prospective cohort study to confirm the results.

## 6. Conclusions

The present study indicated that H-type hypertension and CRP levels increased the risk of RIS, which may be related with inflammation modulation. A further prospective study is necessary to explore the causal relationship among CRP and H-tape hypertension on RIS identified in this study.

## Figures and Tables

**Table 1:** Comparison of general information of patients with first-ever ischemic stroke (FIS) and recurrent ischemic stroke (RIS).

Variable	FIS (*n* = 753)	RIS (*n* = 234)	Total (*n* = 987)	*p* Value
Age (%)				0.004
<60	287 (38.1)	65 (27.8)	352 (35.7)	
≥60	466 (61.9)	169 (72.2)	635 (64.3)	
Gender, *n* (%)				0.001
Male	432 (57.4)	163 (69.7)	595 (60.3)	
Female	321 (42.6)	71 (30.3)	392 (39.7)	
Marital status, *n* (%)				0.679
Single	114 (15.1)	38 (16.2)	152 (15.4)	
Married	639 (84.9)	196 (83.8)	835 (84.6)	
Residence, *n* (%)				0.010
Urban	410 (54.4)	150 (64.1)	560 (56.7)	
Rural	343 (45.6)	84 (35.9)	427 (43.3)	
Risk factors:				
Smoking, *n* (%)	210 (27.9)	85 (36.3)	295 (29.9)	0.018
Alcohol drinking, *n* (%)	171 (22.7)	54 (23.1)	225 (22.8)	0.929
Family history, *n* (%)	118 (15.7)	46 (19.7)	164 (16.6)	0.160
Hypertension	406 (53.9)	158 (67.5)	564 (57.1)	<0.001
Diabetes, *n* (%)	170 (22.6)	69 (29.5)	239 (24.2)	0.036
Coronary heart disease, *n* (%)	97 (12.9)	31 (13.2)	128 (13.0)	0.911
Hyperlipidemia, *n* (%)	481 (63.9)	131 (56.0)	612 (62.0)	0.031
Laboratory findings, mean ± SD				
GLU (mmol/L)	7.07 ± 0.20	7.06 ± 0.34	7.07 ± 0.17	0.987
UA (μmol/L)	286.77 ± 6.15	285.80 ± 12.62	286.54 ± 5.55	0.884
FIB (mg/dL)	305.77 ± 5.27	316.63 ± 12.63	308.34 ± 5.00	0.070
WBC (10^9^/L)	9.60 ± 2.26	11.57 ± 6.84	10.07 ± 2.36	0.487
RBC (10^12^/L)	4.52 ± 0.04	5.63 ± 2.30	4.79 ± 0.55	0.090
PLT (10^9^/L)	235.96 ± 4.49	228.81 ± 9.97	234.26 ± 4.16	0.151
HCY (μmol/L)	19.86 ± 1.07	21.07 ± 2.02	20.15 ± 0.94	0.287
CRP (mg/L)	8.11 ± 1.18	16.94 ± 5.08	10.20 ± 1,51	0.001
HHcy, *n* (%)	667 (88.6)	214 (91.5)	881 (89.3)	0.229
High CRP, *n* (%)	232 (30.8)	89 (38.0)	321 (32.5)	0.046

Note: GLU: Glucose; UA: Uric acid; FIB: Fibrinogen; WBC: White blood cell; RBC: Red blood cell; PLT: Platelet.

**Table 2:** Comparison of hypertension and hypertension with high homocysteine (HHcy) between FIS and RIS groups.

Variable	FIS (*n* = 753)	RIS (*n* = 234)	χ^2^ Value	*p* Value
Hypertension and HHcy (%)			14.989	0.002
Normal	46 (88.5)	6 (11.5)		
Simple hypertension	40 (74.1)	14 (25.9)		
Simple HHcy	301 (81.1)	70 (18.9)		
H-type hypertension	366 (71.8)	144 (28.2)		

Note: Normal: Patients without hypertension and HHcy; Simple Hypertension: Patients with hypertension without HHcy; Simple HHcy: Patients with HHcy without hypertension; H-tape Hypertension: Patients with hypertension and HHcy.

**Table 3:** Multivariate logistic regression analysis of factors related to RIS.

Factor	OR ^a^	95% CI	*p*
Age			
<60	Reference		
≥60	1.576	1.125–2.207	0.008
Gender			
Female	Reference		
Male	1.935	1.385–2.704	<0.001
Diabetes			
No	Reference		
Yes	1.463	1.037–2.064	0.030
CRP (mg/L)	1.013	1.006–1.019	<0.001
H-type			
Normal	Reference		
Simple Hypertension	3.370	1.115–10.183	0.031
Simple HHCY	1.843	0.710–4.782	0.209
H-type Hypertension	2.990	1.176–7.600	0.021

Note: OR: Odds ratio; 95% CI: 95% confidence interval; normal: Patients without hypertension and HHcy; simple Hypertension: Patients with hypertension without HHcy; simple HHcy: Patients with HHcy without hypertension; H-tape Hypertension: Patients with hypertension and HHcy. ^a^ Adjusted for all other variables included in the table.

## Abbreviations

CRP	C reactive protein
RIS	Recurrent ischemic stroke
FIS	First-ever ischemic stroke
Hcy	Homocysteine
